# Magnetotelluric evidence for highly focused mantle melting along the ultraslow-spreading Gakkel Ridge, Arctic Ocean

**DOI:** 10.1093/nsr/nwaf077

**Published:** 2025-02-28

**Authors:** Tao Zhang, Jiabiao Li, Weiwei Ding, Fansheng Kong, Yinxia Fang, Xiongwei Niu, Jie Jiang, Zhiteng Yu, Pingchuan Tan, Zhongyan Shen, Chunguo Yang, Qiuci Sun, Zhezhe Lu, Bo Yang, Yanan Liu, Yejian Wang, Yunsheng Zhao

**Affiliations:** State Key Laboratory of Submarine Geoscience, Second Institute of Oceanography, Ministry of Natural Resources, Hangzhou 310012, China; State Key Laboratory of Submarine Geoscience, Second Institute of Oceanography, Ministry of Natural Resources, Hangzhou 310012, China; State Key Laboratory of Submarine Geoscience, Second Institute of Oceanography, Ministry of Natural Resources, Hangzhou 310012, China; State Key Laboratory of Submarine Geoscience, Second Institute of Oceanography, Ministry of Natural Resources, Hangzhou 310012, China; State Key Laboratory of Submarine Geoscience, Second Institute of Oceanography, Ministry of Natural Resources, Hangzhou 310012, China; State Key Laboratory of Submarine Geoscience, Second Institute of Oceanography, Ministry of Natural Resources, Hangzhou 310012, China; Key Laboratory of Ocean and Marginal Sea Geology, South China Sea Institute of Oceanology, Chinese Academy of Sciences, Guangzhou 510301, China; University of Chinese Academy of Sciences, Beijing 100049, China; State Key Laboratory of Submarine Geoscience, Second Institute of Oceanography, Ministry of Natural Resources, Hangzhou 310012, China; State Key Laboratory of Submarine Geoscience, Second Institute of Oceanography, Ministry of Natural Resources, Hangzhou 310012, China; State Key Laboratory of Submarine Geoscience, Second Institute of Oceanography, Ministry of Natural Resources, Hangzhou 310012, China; State Key Laboratory of Submarine Geoscience, Second Institute of Oceanography, Ministry of Natural Resources, Hangzhou 310012, China; State Key Laboratory of Submarine Geoscience, Second Institute of Oceanography, Ministry of Natural Resources, Hangzhou 310012, China; State Key Laboratory of Submarine Geoscience, Second Institute of Oceanography, Ministry of Natural Resources, Hangzhou 310012, China; Key Laboratory of Ocean and Marginal Sea Geology, South China Sea Institute of Oceanology, Chinese Academy of Sciences, Guangzhou 510301, China; State Key Laboratory of Submarine Geoscience, Second Institute of Oceanography, Ministry of Natural Resources, Hangzhou 310012, China; State Key Laboratory of Submarine Geoscience, Second Institute of Oceanography, Ministry of Natural Resources, Hangzhou 310012, China; Yangtze Delta Region Institute (Huzhou), University of Electronic Science and Technology of China, Huzhou 313000, China

**Keywords:** Arctic Ocean, mid-ocean ridge, magnetotellurics, geodynamics, lithosphere

## Abstract

It is well known that ultraslow-spreading mid-ocean ridges display significant variations in axial magmatism and tectonics. Yet, the processes governing mantle melting and melt transport remain a subject of ongoing debate. A key limitation has been the lack of contrasting observations of mantle melting beneath axial segment centers versus segment ends, particularly through electromagnetic methods, which are highly sensitive to partial molten mantle. Here, we present the first one-dimensional magnetotelluric observation conducted along the ultraslow-spreading Gakkel Ridge in the Arctic Ocean. Our findings reveal prominent low-resistivity zones at depths of 20–45 km beneath segment centers, which are indicative of shallow melting zones. We propose that the robust magma supply and associated repeated magma intrusions lead to a thin thermal lithosphere and associated shallow mantle melting. In contrast, such electrical resistivity anomalies are absent at comparable depths beneath the magma-poor deep valley, where the electrical lithosphere extends to depths of >50 km. The extremely thick lithosphere restricts mantle melting to greater depths and facilitates melt migration toward adjacent segment centers. Our study highlights the critical role of highly variable lithospheric thickness in regulating melting depth and focusing melt flow along ultraslow-spreading ridges. We propose that the significant variation in lithospheric thickness and the associated focused melting result in the recently observed highly variable crustal thickness along the Gakkel Ridge.

## Teaser

This paper reports the first one-dimensional magnetotelluric experiment conducted along the ultraslow-spreading Gakkel Ridge in the Arctic Ocean. The findings reveal significant variations in lithospheric thickness along the ridge axis, which may explain the previously observed highly focused magmatism there.

## INTRODUCTION

Magmatic accretionary ridge segments are second-order segments [1] and are considered the basic unit of magmatic accretion along a mid-ocean ridge [2]. Slow- and ultraslow-spreading mid-ocean ridges are characterized by significant variations in along-axis crustal thickness, with notable thinning from segment centers to the ends. To explain this highly variable magmatism, several models have been proposed, addressing processes from deep to shallow levels. These include focused deep mantle upwelling, melt aggregation along the base of the lithosphere, and tectonic-magmatic instability-induced melt partitioning [3–7]. Lin *et al.* [3] suggested that each second-order ridge segment operates as a mantle upwelling cell, with the highest mantle upwelling velocity at the segment center. Particularly in low-viscosity, wet melting zones, segment-scale convection may concentrate mantle upwelling toward the centers, enhancing melt production [5]. Alternatively, it has been proposed that the lithospheric base acts as a permeability barrier, guiding melt migration from segment ends to the center along its slope [4,7]. Geochemical analyses of volcanos at the segment center along the ultraslow-spreading Southwestern Indian Ridge (SWIR) support this focused melting model [8]. The recent numerical study suggests that reduced melt supply, detachment faults, and variations in lithospheric thickness may drive tectono-magmatic instability and associated melt partitioning along an ultraslow-spreading ridge axis [6]. Liu and Buck [9] further suggest that concentrated heat from focused melt flow causes localized lithospheric thinning at the segment center of a slow- or ultraslow-spreading ridge, meeting the conditions necessary for the formation of stable melt lens [10]. This mechanism could also account for the pronounced variation in magma accretion along an ultraslow-spreading ridge axis.

**Figure 1.**
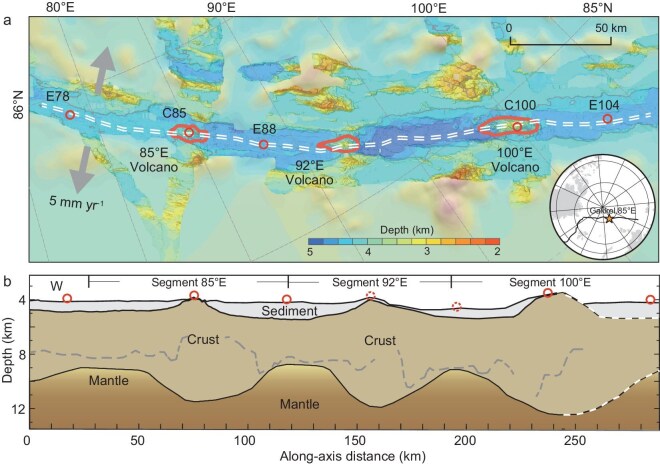
(a) Bathymetry and magnetotelluric (MT) survey layout. Red circles mark the MT sites. Red lines depict the volcanic centers with 3500 m contours, and the double dashed line indicates the ridge axis. The inset shows the position of the survey area. (b) Along-axis crustal structure. The boundaries between water, crust, and mantle are derived from seismic and gravity data [15]. Red dashed circles indicate unrecovered MT sites. The dashed gray lines denote the boundary between seismic layers 2 and 3. East of 100°E, the crustal structure remains unconstrained and is outlined with dashed lines.

The magnetotelluric (MT) survey provides an effective method for investigating mantle melting and testing these hypotheses. However, previous MT observations at ultraslow-spreading ridges have been limited to across-axis profiles, such as those at the Mohns and Knipovich ridges [11,12]. The Gakkel Ridge, the world's slowest spreading ridge, shows substantial variability in magmatism [13–15], making it an ideal location to study the processes of mantle melting, melt transport, and the associated intermittent magmatism. Along the eastern Gakkel Ridge (Fig. 1), three evenly spaced volcanic centers are located at 85°E, 92°E, and 100°E, aligning with the centers of three magmatic segments as defined by the crustal structure [15]. Here, we present MT data from two segment centers and three segment ends, where severe sea-ice conditions made such ocean-bottom observations very challenging. Our findings reveal low-resistivity anomalies at the depth of the anhydrous melting zone beneath the two segment centers, contrasted by extremely thick electrical lithosphere at segment ends. We propose that the significantly variable lithospheric base drives the highly focused melting at the Gakkel Ridge.

## GEOLOGICAL SETTINGS

The spreading rate of the Gakkel Ridge decreases from 14 mm yr^−1^ near the Fram Strait in the west to ∼7 mm yr^−1^ in the Laptev Sea to the east, making the eastern section the slowest-spreading mid-ocean ridge in the world. It is characterized by punctuated magmatism and highly variable crustal structure along the ridge axis [2,14,15]. Volcanic centers at the Gakkel Ridge may persist for a few million years [16].

The 85°E volcanic center has recently experienced volcanic eruptions [17], earthquakes [18], and hydrothermal activities [17,19]. Active-source ocean-bottom seismometer array surveys along the ridge axis indicate an average crustal thickness of 5.5 km in the study area [15], significantly thicker than previous estimates of ∼2 km [20,21]. Crustal thickness at the volcanic centers reaches up to 7.5 km at 85°E and 8.9 km at 100°E, while it attenuates to 3.3–4.5 km at segment ends. Geochemical analyses suggest an average melting degree of 3.7% beneath the 85°E volcanic center, with the melting zone extending from depths of 25 to 40 km [22].

## DATA AND INVERSION

During the Joint Arctic Scientific Mid-Ocean Ridge Insight Expeditions (JASMInE) in 2021 [23] and JASMInE-2 in 2024, we deployed seven MT instruments along the Gakkel Ridge, spanning 78°–104°E in areas densely covered by sea ice (Fig. 1). Five instruments were successfully recovered. Of these, sites C85 and C100 are positioned at the volcanic centers of the 85°E and 100°E segments, respectively, while E78, E88, and E104 are situated near the segment ends. The water depths at these sites range from 3.7 to 4.4 km. Each MT instrument recorded data on the seafloor for 12–25 days (see Table S1), providing sufficient time to capture the electrical structure within the upper mantle melting zone (Fig. S1).

**Figure 2.**
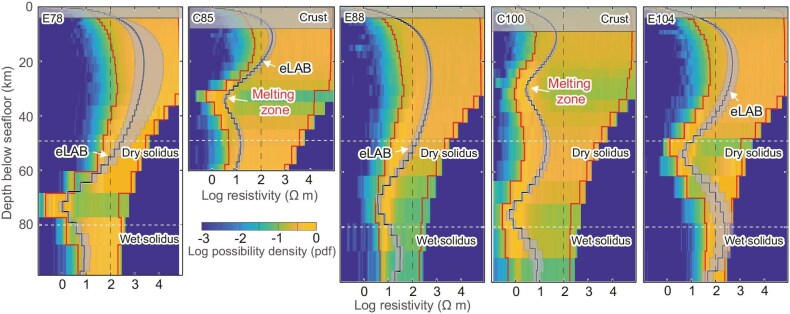
Inverted 1-D electrical mantle structure. Blue lines represent the results of Occam inversion used for interpretations in this study. The shaded regions depict the maximal values of the five closest inversion iterations (Fig. S7). The shaded areas are narrow in regions of relatively low resistivity, especially within the shallow low-resistivity zones at sites C85 and C100. Since our focus is on low resistivities, those narrow-shaded areas indicate that once the inversion result stabilizes, the specific choice of inversion result has minimal impact on our interpretations. The Posterior model parameter (PDF) uncertainty is estimated from resistivity data using trans-dimensional Bayesian inversion [27–30] and shown in color image. Warmer colors indicate regions with higher probability. The red lines delineate the 90% credible interval. Note that the black lines, especially the low-resistivity part, show good agreement with the warm-colored, high-probability zones of the PDF. Depths for the solidi of dry and wet (245 ppm H_2_O) peridotites with an assumed mantle potential temperature (T_p_) of 1360°C are indicated by dashed lines. Based on the peridotite solidi proposed by Sarafian *et al.* [31] and the inverted electrical resistivities here, we estimate the T_p_ in the study area to be approximately 1360°C. This estimated T_p_ is higher than the apparent T_p_ derived from the major-element compositions of basalts [15], which often lead to lower values due to the consideration of mantle cooling during melting [32]. At the depth of hydrous mantle melting, the effective electrical resistivity of the mantle could be further disproportionately reduced by the small amount of carbonatite melts at this depth [33]. Following the definitions provided by Johansen *et al.* [12], we designated electrical resistivity values of 25 Ω m and 100 Ω m as the boundaries for the melting zone and the base of the electrical lithosphere (eLAB), respectively.

Dimensionality analysis conducted for the five MT sites (see Methods) confirmed that the signals display 2-D characteristics over 215–3400 s (Fig. S5). Due to compass failures, orientation data for the three MT instruments (C85, E88, C100) deployed in 2021 are absent. However, the MT fields were rotated to represent electric field models aligned parallel (TE) and perpendicular (TM) to the strike of the Gakkel Ridge, as expected in a 2-D structure. We thus analyzed invariable TE and TM impedances, ensuring that the impedance tensor's diagonal elements become zeros as in an ideal 2-D case (see Methods) [24–26]. Our analysis focuses on the TE polarization mode to characterize the electrical structure along the ridge axis (Fig. 2).

Given the limited station count and wide spacing between them, we applied a 1-D Occam inversion method [34–36] to infer the electrical structure of the upper mantle. Except for site C85, which has a root mean square misfit of 1.8, the other sites have root mean square misfits ranging from 1.1 to 1.2. We further evaluated the robustness of the inversion results by conducting uncertainty quantification using the trans-dimensional Bayesian inversion [27–30]. The electrical resistivities derived from the 1-D Occam inversion align well with high-probability regions from the Bayesian approach, confirming the reliability of our results (Fig. 2). We then used the results of 1-D Occam inversion for interpretations, as this approach is widely applied in similar tectonic settings [35,36].

## RESULTS AND DISCUSSION

### Melting zones beneath segment centers

**Figure 3.**
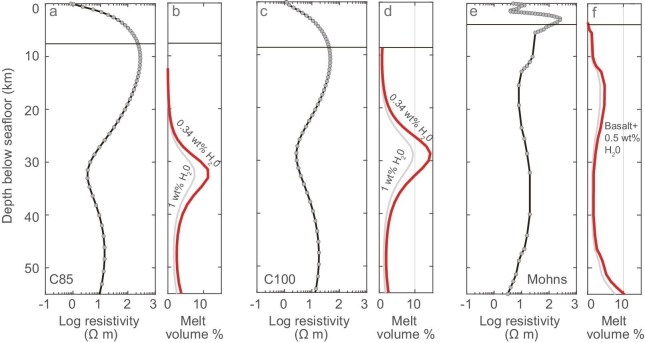
Electrical resistivity and associated melt contents at sites C85 (a, b), C100 (c, b), and the Mohns Ridge (e, f) [12]. The water content of melt in the study area is under the saturation point [37].

Low resistivities (<25 Ω m) are observed at depths of 20–45 km below the seafloor at the two sites situated on volcanic centers of segments (Fig. 2). At the 85°E volcanic center, these depths align with the melting zones estimated from petrological data (∼25–40 km) [22]. With a water content of the primary magma around 0.34 wt% in this region [15], melting fractions could reach up to 14% at C100 and 11% at C85, producing the observed low resistivities (Fig. 3 and Fig. S10). The average resistivity, which is less model-dependent, is ∼12 Ω m within the low-resistivity zone of site C85. This average value could be attributed to ∼4.0% melt content in the mantle, aligning closely with an average degree of melting (3.7%) suggested by analyses of glasses and inclusions at the 85°E volcanic center [22].

The depth and extent of mantle melting are primarily governed by mantle temperature and composition. Due to conductive heat loss from above, the depth at which melting ceases increases as the spreading rate decreases beneath a ridge axis [21,38]. At the segment centers in the study area, the upper boundary (defined as 25 Ω m) of the melting zone is relatively shallow (∼20–25 km below the seafloor), indicating an unexpectedly thin thermal lithosphere. This depth is also comparable to that observed beneath the segment center of the ultraslow-spreading Mohns Ridge (Fig. 3) [12]. As we will discuss further below, the mantle melting at segment ends is suppressed to a much greater depth (Fig. 2). Given the same spreading rate and thus the similar effects of conductive cooling, the variable depths of the upper boundary of the mantle melting zones in the study area suggest that factors beyond T_p_ and conductive cooling also play a role in modulating the final melting depth. Recent numerical simulations indicate that focused melt flow [9] and repeated magma intrusions [39] elevate isotherms at the level of magma lens of ultraslow-spreading ridges. Given the enhanced crustal thickness (7.5 km and 8.9 km, respectively) and the associated intensive magmatism at C85 and C100, we propose that the substantial volume of melt may provide excess heat, counteracting conductive cooling and resulting in a relatively thin lithosphere and shallow final melting depth.

### Thick electrical lithosphere beneath segment ends

In contrast to the prominent low-resistivity zones beneath segment centers, sites at the ends of magmatic segments show high resistivities (>100 Ω m) down to ∼50 km, indicating a significantly thick electrical lithosphere (Fig. 2). Beneath this thick lithosphere, low-resistivity layers have been observed at ∼50–85 km depth. With a measured water content of 245 ppm [15] and an assumed T_p_ of 1360°C of the oceanic upper mantle, the depths of dry and wet solidi are estimated to be ∼50 km and 80 km [31], respectively (Fig. 2). The absence of low resistivity above the dry solidus implies that the anhydrous melting is either suppressed or that any generated melt is efficiently transported away. The deep low-resistivity layers are likely associated with the wet melting zone.

Enhanced conductive cooling and the thick lithosphere at the three segment ends could restrict steady-state anhydrous mantle melting from above. Observations from the ultraslow-spreading Knipovich Ridge and SWIR [40] show that the maximum depths of earthquakes—commonly associated with the ∼680°C isotherm—are 15–25 km below the seafloor. Assuming the 680°C isotherm lies at ∼20 km depth and a constant geotherm gradient below, mantle melting would commence below ∼35–40 km, leaving a deep and relatively low-degree of mantle melting. Since MT data can detect interconnected low-degree melting [41], the absence of low resistivity above 50 km at segment ends in the study area suggests that any deep, low-degree melts must be transported efficiently to adjacent segment centers, frozen in the thick lithosphere, or intruded to the surface.

The significantly thick crust at the segment centers of ultraslow-spreading ridges supports the idea of along-axis melt focusing from segment ends to centers [15,42]. With lithospheric thickness at ∼50 km at segment ends, ∼20 km at centers, and spacing of ∼40 km between them, the lithospheric base has a steep slope (∼0.75), much steeper than the slope (0.15) proposed for efficient melt migration [43]. Numerical models also suggest that the along-axis lithospheric permeability barrier should be strong at an ultraslow-spreading ridge [43], facilitating the aggregation of low-degree melts from segment ends to neighboring centers along the slope. Therefore, we propose that a large part of the melt produced at segment ends migrates to segment centers along the steep lithospheric base. This process could be efficient, as the melt migration rate is a few orders higher than the spreading rate at an ultraslow-spreading ridge [44,45].

### Highly variable magmatism along the Gakkel Ridge

**Figure 4.**
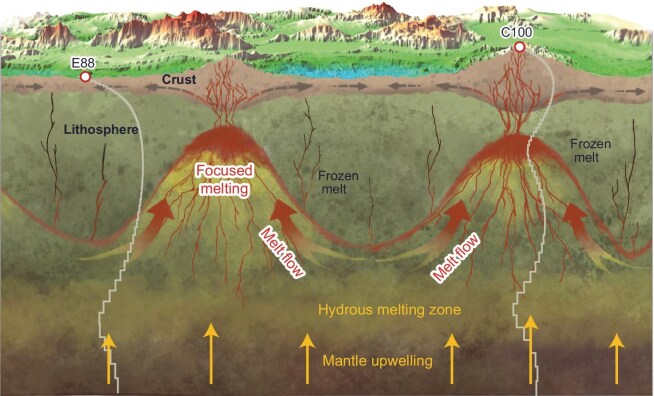
Schematic 3-D along-axis section illustrating the role of modulation by the lithosphere on melt focusing and generation. The top surface is a map view of the multibeam bathymetry in Fig. 1. The lithospheric base is shallow near segment centers and could extend to the depth of the wet melting zone at segment ends. The highly variable lithosphere promotes melt migration from segment ends to centers. The focused melt may be redistributed to segment ends at a crustal level through dike propagations (gray arrows) [46] or lava flows [47]. The mantle upwelling in the region of wet melting may also be more pronounced at segment centers compared to segment ends [5]. Two magnetotelluric sites and associated 1-D resistivity profiles are also shown here (See Fig. 2 for details). The entire section spans ∼180 km in length, with the vertical axis not drawn to scale.

Together, the along-axis variations in the extent of mantle melting at the Gakkel Ridge reflect the interactions among lithospheric thermal structure, melt production, and melt transportation (Fig. 4). At segment ends, a thick lithosphere impedes mantle melting and facilitates the generated melt migration toward the adjacent segment center along the permeability barrier near the base of the lithosphere. At segment centers, enhanced magma supply and repeated magma intrusions attenuate the lithospheric thickness, which, in turn, promotes melt production and further concentrates melt flow. This self-sustaining mechanism may account for the persistence of volcanic centers here and at other ultraslow-spreading ridges [16].

Our MT observations then evidence that the extremely variable along-axis crustal thickness, ranging from 3.3 to 8.9 km as identified by seismic studies [15], results from the significant variation in lithospheric thickness and the associated focused melting (Fig. 4). While the relatively thin crust at the segment ends of the Gakkel Ridge may partly arise from melt extraction occurring directly beneath the crust, the absence of significant melt zones beneath segment ends suggests that crust formation in these regions is dominated by redistributions of the focused melt, such as dike propagation within the crust [46] or lava flow on the seafloor [47]. This shallow-level crust-building model is consistent with the observed absence of gabbros at segment ends, as indicated by seismic data from the study area [15] and other ultraslow-spreading ridges [14,48,49]. At the 85°E volcanic center, high degrees of mantle melting correlate well with thick crust and low seismic velocities in the lower crust, both indicative of elevated temperatures and melt accumulation [15].

Remarkably, the amplitudes of electrical resistivity at the Gakkel Ridge are comparable to those at the slow-spreading northern Mid-Atlantic Ridge (Fig. S11) [35], where crustal thickness reaches up to 7.5 km at a segment center [50]. Given the spreading rate at the northern Mid-Atlantic Ridge is ∼2 times that of our study area, the similar electrical resistivity amplitudes and associated melting degrees between these two ridges suggest that the high-extent mantle melting and thick crust at the Gakkel Ridge are not governed solely by the spreading rate and associated passive mantle upwelling. Other factors, such as mantle heterogeneity and active mantle upwelling, must play critical roles in mantle melting. As the spreading rate decreases to 10 mm yr^−1^, active mantle upwelling leads to significantly higher mantle upwelling velocities (approximately twice as fast), greater melting degrees (∼1.5 times higher), and substantially thicker crust (∼2.5 times thicker) compared to predictions of the passive mantle upwelling model [15]. Therefore, active and buoyant mantle upwelling, combined with a relatively fertile mantle composition, provides the most plausible explanation for the high melting degree and thick crust observed at the Gakkel Ridge.

## CONCLUSIONS

Data from five MT sites along the Gakkel Ridge reveal the electrical mantle structure, showing highly focused mantle melting from segment ends to centers. Low resistivities at 20–45 km depths beneath segment centers indicate melting zones beneath a thermally thinned lithosphere. In contrast, the thick electrical lithosphere observed beneath segment ends reflects enhanced conductive cooling associated with the ultraslow-spreading rate. We propose that the significant along-axis variations in magmatism and associated crustal thickness at ultraslow-spreading ridges are governed by a highly variable lithospheric thickness shaped by conductive cooling, melt production, and melt transport.

## METHODS

We used marine electromagnetic receivers Micro-OBEM developed by China University of Geosciences (Beijing) for data acquisition [51]. The robust transfer function code [52] was used on the time series of the raw data to obtain the apparent resistivity and phase (Fig. S1). We demonstrate that the nonplanar electromagnetic waves that did not meet the MT assumption were negligible, as verified by comparing MT response functions during high and low diurnal signal intervals following the methods of Hill *et al.* [53] (Figs S2–S4). Dimensional analyses were operated using the open-source software MTpy [54,55]. Sites C85 and C100, located at volcanic centers, displayed 2-D characteristics for periods <3400 s and transitioned to 3-D at more extended periods. Sites E78, E88, and E104, on the segment ends, showed 1-D characteristics for periods between 500–2100s, transitioning to 2-D and 3-D at longer periods.

We utilized two rotation invariants to compact the four elements of the tensor into TE and TM impedances for sites C85, E88, and C104 (Fig. S6). This method ensures that the diagonal elements become zero, as in the ideal 2-D case, by solving a quadratic equation [24]. It allows us to calculate each site's invariant TE and TM consistently. It has been proven effective even in cases of inconsistent strike directions between periods or a lack of orientation due to compass failure [24,56].

For data inversion (Figs S7–S8), we used the Occam method [34] for 1-D inversion and performed uncertainty quantification with a trans-dimensional Markov chain Monte Carlo (MCMC) algorithm [27]. Geological models were based on seismically determined crustal structures, with seawater and underlying half-space layers included in the inversion processes [15]. The error floors for apparent resistivity and phase were set at 5% and 1.43°, respectively. Results from both the Occam and MCMC methods showed that high-probability areas align closely with low-resistivity zones (Fig. 2).

The theoretical thermal structure along the ridge axis was determined by combining the conductive cooling and adiabatic upwelling models [12,57]. The theoretical thermal structure is constrained by the inversion results and the associated temperature model of dry peridotite (SEO3) [58]. According to the obtained temperature profiles, the melting degree at different depths of the mantle of the Gakkel Ridge (Fig. S10) was calculated based on the resistivity mixing model for polyphase materials (dry peridotite and molten peridotite) [59] and the mid-ocean ridge basalts model at varying water contents [60].

## Supplementary Material

nwaf077_Supplemental_File
