# Supplementary material for: Magnetotelluric evidence for highly focused mantle melting along the ultraslow-spreading Gakkel Ridge, Arctic Ocean
Source: Natl Sci Rev. 2025 Feb 28;12(5):nwaf077. doi: 10.1093/nsr/nwaf077 (PMC12010957; doi:10.1093/nsr/nwaf077)
Supplement: nwaf077_Supplemental_File [file nwaf077_supplemental_file.docx]

Supplementary Materials for

## **Magnetotelluric evidence for highly focused mantle melting along the ultraslow-spreading Gakkel Ridge, Arctic Ocean**

Tao Zhang^1^, Jiabiao Li^1*^, Weiwei Ding^1^, Fansheng Kong^1^, Yinxia Fang^1^, Xiongwei Niu^1*^, Jie Jiang^2,3^, Zhiteng Yu^1^, Pingchuan Tan^1^, Zhongyan Shen^1^, Chunguo Yang^1^, Qiuci Sun^1^, Zhezhe Lu^1^, Bo Yang^2^, Yanan Liu^1^, Yejian Wang^1^, Yunsheng Zhao^4^

1 State Key Laboratory of Submarine Geoscience, Second Institute of Oceanography, Ministry of Natural Resources, Hangzhou 310012, China

2 Key Laboratory of Ocean and Marginal Sea Geology, South China Sea Institute of Oceanology, Chinese Academy of Sciences, Guangzhou 510301, China

3 University of Chinese Academy of Sciences, Beijing 100049, China

4 Yangtze Delta Region Institute (Huzhou), University of Electronic Science and Technology of China, Huzhou 313000, China

*Corresponding author: jbli@sio.org.cn; xwniu@sio.org.cn

**Contents of this file**

Supplementary Data and Methods

Figs. S1 to S11

Table S1

## Supplementary References

## **Data and Methods**

**MT instruments and raw data**

We used the marine electromagnetic receivers developed by the China University of Geosciences (Beijing) (Micro-OBEM) for data collection. Each Micro-OBEM consists of two sets of low-noise, horizontal, orthogonal silver-silver chloride (Ag-AgCl) electrodes and induction coil sensors, where the length of the electrode dipole is 8 m [1]. In August 2021 and September 2024, we successfully recovered three instruments from sites C85, E88, and C100 and two from sites E78 and E104. The instruments were deployed on the seafloor for 12 to 25 days. During the period, the instrument at site C85 functioned normally for only 7 days; the others successfully collected data throughout their deployment (see Table S1). Unfortunately, due to compass malfunctions, orientation information was lost for the three MT instruments (C85, E88, C100) deployed in 2021. We applied robust transfer function algorithms [2] to the raw time series data from the marine MT measurements, resulting in high-quality apparent resistivities and phases for the five sites. The original apparent resistivity and phase data can be found in Fig. S1.

**Table S1.** Effective collection times for the MT sites

| Sites | Starting Time | Ending Time | Effective Time (days) |
| --- | --- | --- | --- |
| E78 | 23-Aug 202412:36:02 | 03-Sep 2024 12:41:10 | 12.0 |
| C85 | 16-Aug 2021 04:57:05 | 23-Aug 2021 07:02:05 | 7.1 |
| E88 | 12-Aug 2021 02:08:26 | 28-Aug 2021 07:43:26 | 16.2 |
| C100 | 7-Aug 2021 00:42:52 | 1-Sep 2021 05:52:52 | 25.2 |
| E104 | 21-Aug 2024 21:20:49 | 14-Sep 2024 20:07:18 | 23.9 |


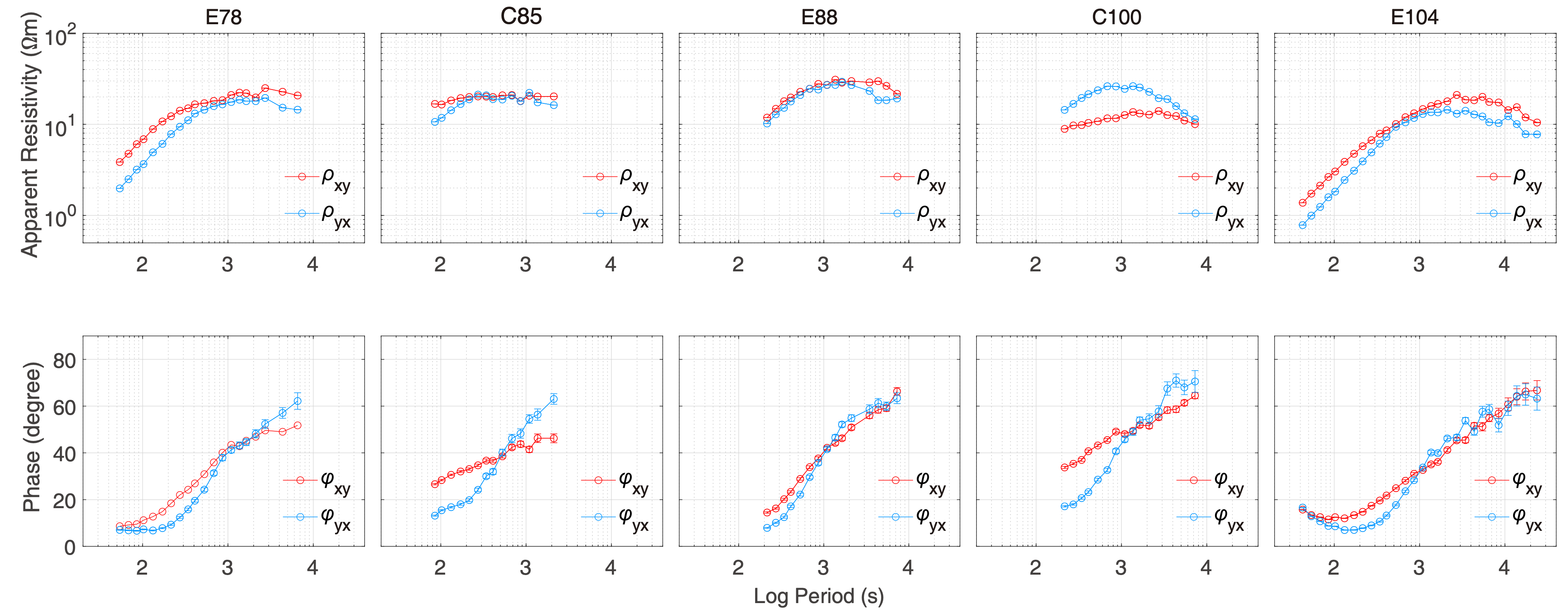


**Figure S1.** Raw apparent resistivity and phase.

**Non-planar source effects analysis**

The validity of the plane wave assumption may be compromised when collecting magnetotelluric data in polar regions [3]. To evaluate the impact of source effects on the field data, we analyzed the planetary A-index (Ap) magnetic activity indices during the data collection, as well as the temporal spectral strength of the electromagnetic field. The Ap indices presented in Fig. S2 (top panel) primarily indicate low activity levels, categorized as Quiet and Unsettled, except on August 28, 2021, when activity levels escalated to Active and Minor Storm. In contrast, the magnetic activity index peaked on September 12, 2024, near 130, indicating a Strong Storm (bottom panel in Fig. S2).

Further analysis of the spectral strength of electric and magnetic fields indicate that the Minor Storm on August 28, 2021, and the Strong Storm on September 12, 2024, had minimal impact on the time series data. However, disturbances were noted in the electric field at site C100, as illustrated by the dashed rectangular box in Fig. S3. We categorized these disturbed data segments and those associated with the Strong Storm at site E104 as "bad records" during processing with the robust transfer function code [2]. The comparison of results before and after this classification for sites C100 and E104, shown in Fig. S4, reveals that the interferences had a limited effect on data segments shorter than 8000 seconds.


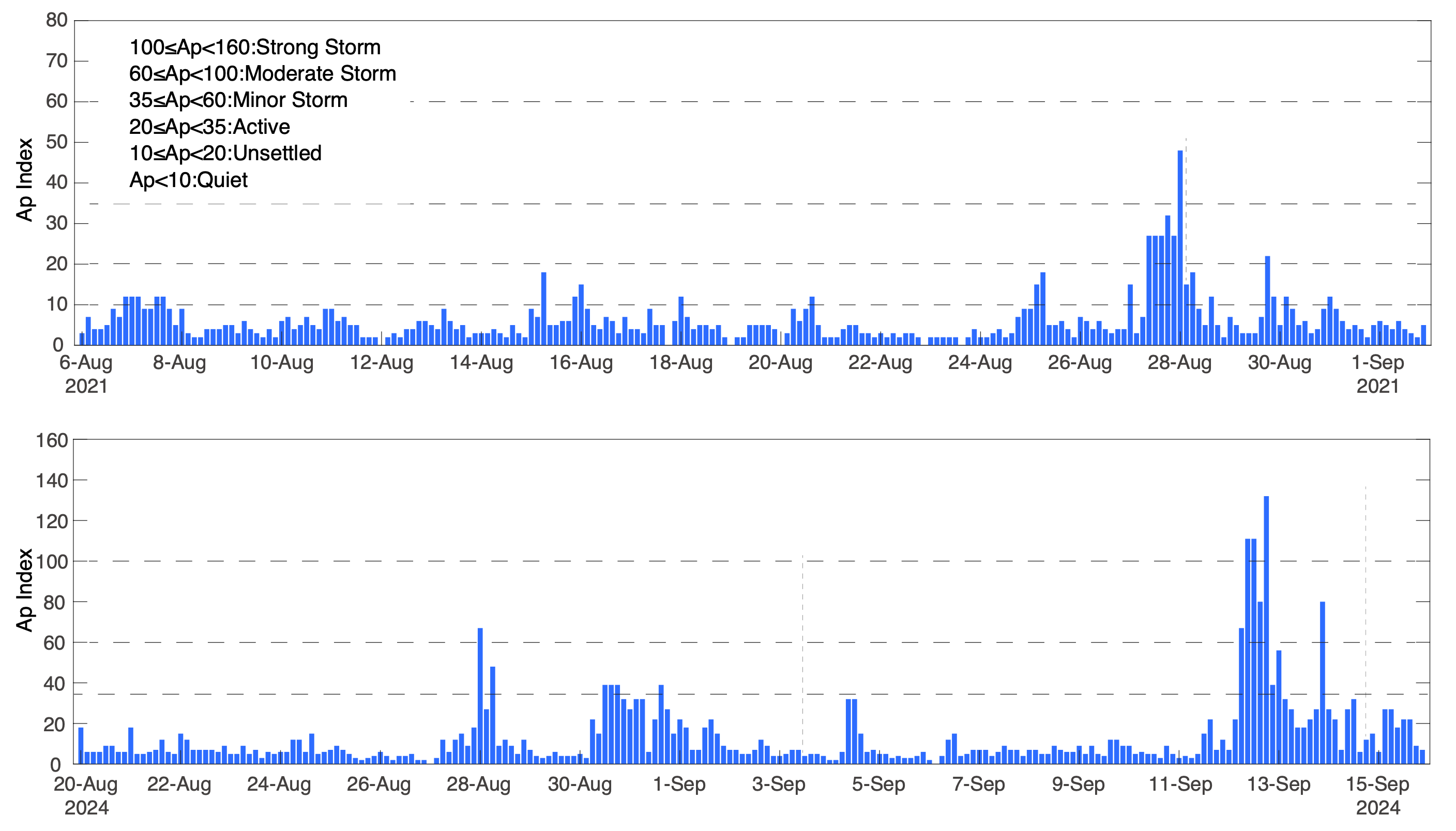


**Figure S2.** The Ap magnetic activity indices were recorded eight times daily, with each bar representing the average Ap indices over three hours. Data were obtained from https://kp.gfz-potsdam.de/en.


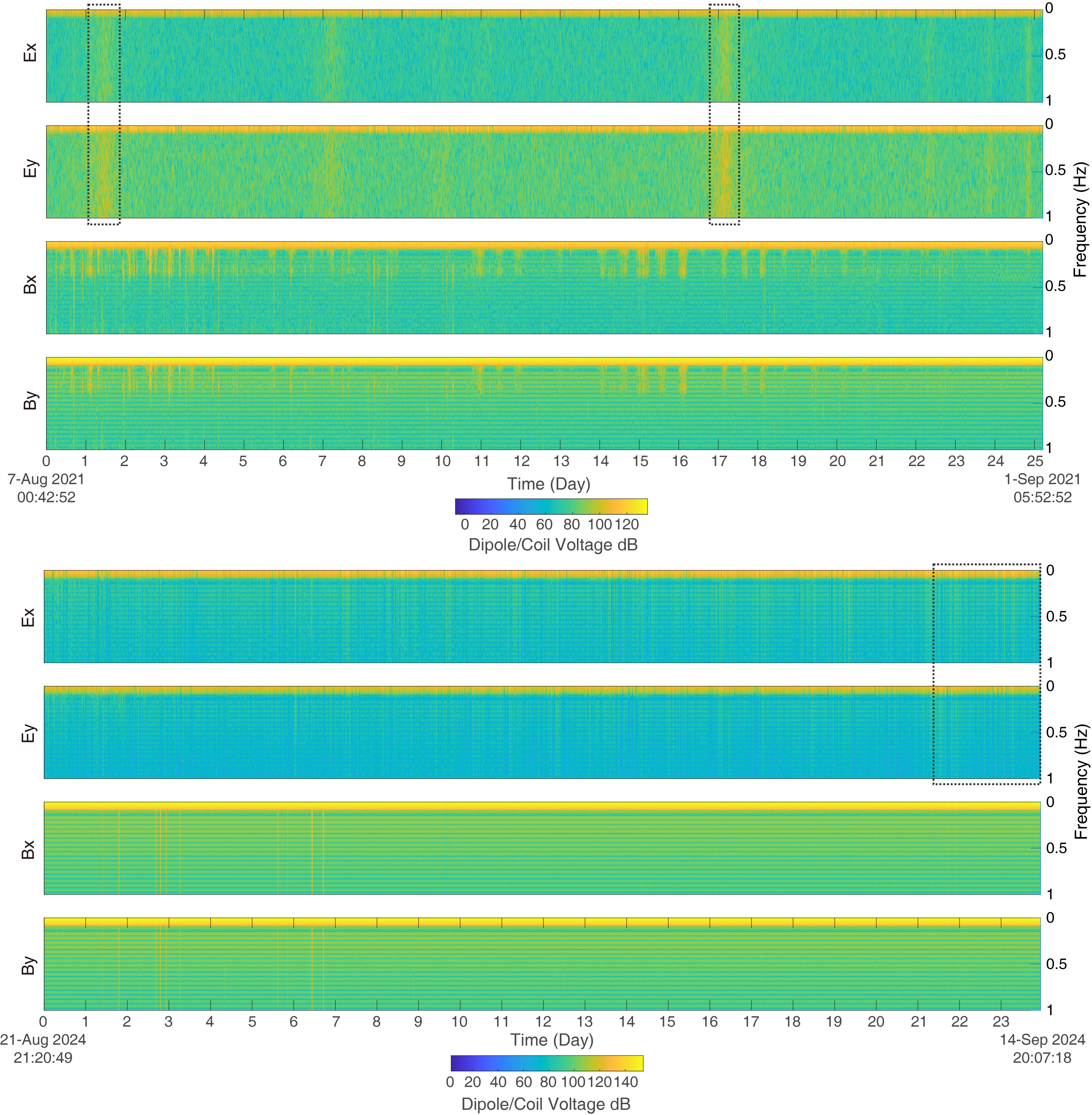


**Figure S3.** Spectral strength of the electric and magnetic fields for sites C100 (upper) and E104 (lower). The periods marked with dashed rectangular boxes indicate electrical current disturbances and the periods associated with the Strong Storm, were classified as 'bad records' during data processing.


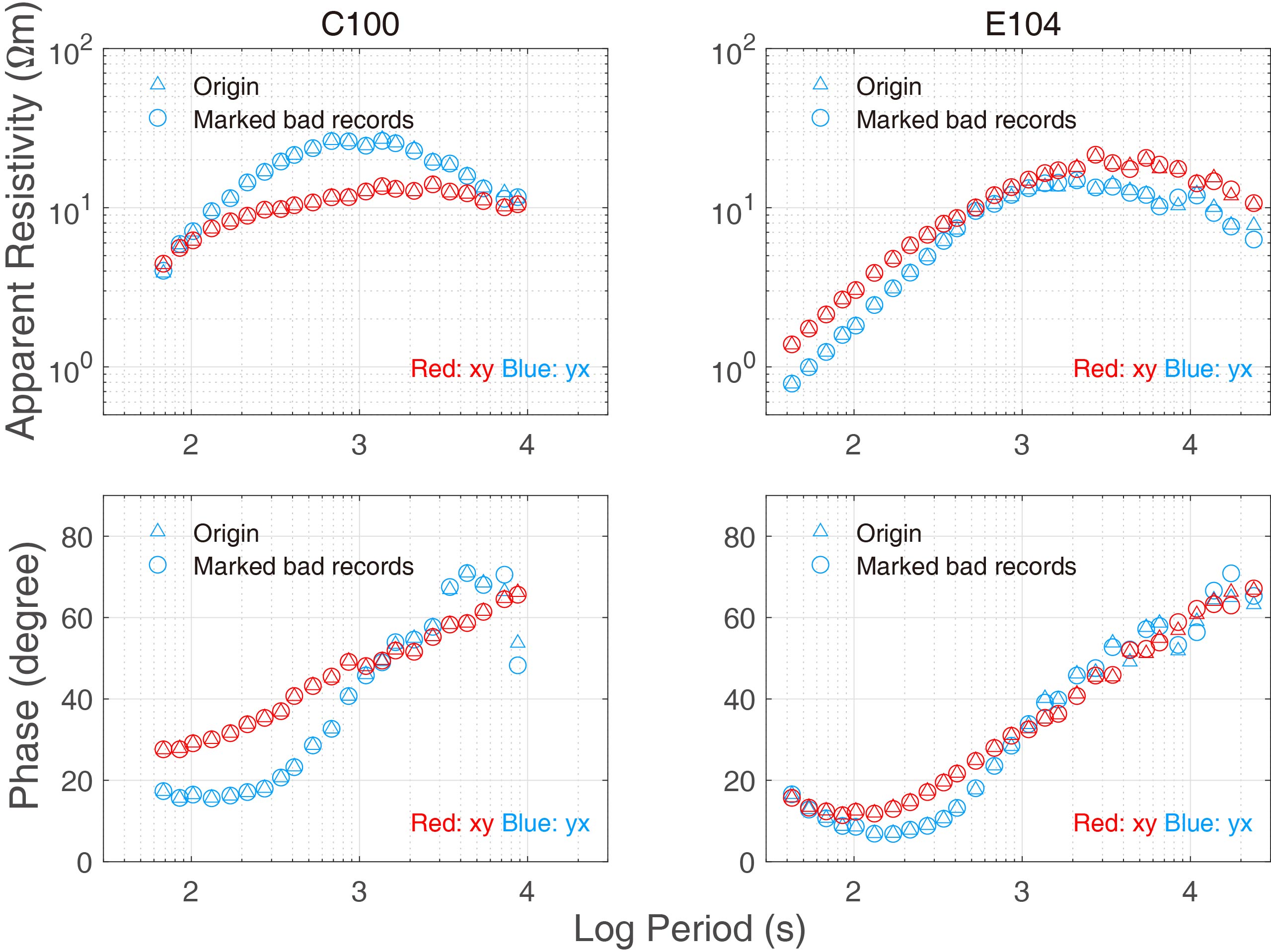


**Figure S4.** Comparison of apparent resistivity and phase results before and after marking "bad records" using the robust transfer function code for sites C100 and E104.

**Dimensionality analysis**

We conducted dimensional analyses of all the MT sites using the open-source software MTpy [4,5]. The dimensionality at each frequency for every site can be assessed through the phase tensor ellipse, as shown in Fig. S5. When the skew angle of the phase tensor ellipse exceeds 3 degrees, the geological structure tends to exhibit 2-D characteristics; as the angle increases, the 3-D features become more pronounced [4,6–8]. Sites C85 and C100, located at the volcanic centers, display geological structures that resemble 2-D features but tend to exhibit 3-D characteristics for periods longer than 3400 seconds. In contrast, sites E78, E88, and E104 at the volcanic ends show approximately 1-D characteristics during periods ranging from 500 to 2100 seconds. For periods longer than 2100 seconds, these sites tend to demonstrate 2-D structural features, transitioning toward 3-D structures as the period increases.


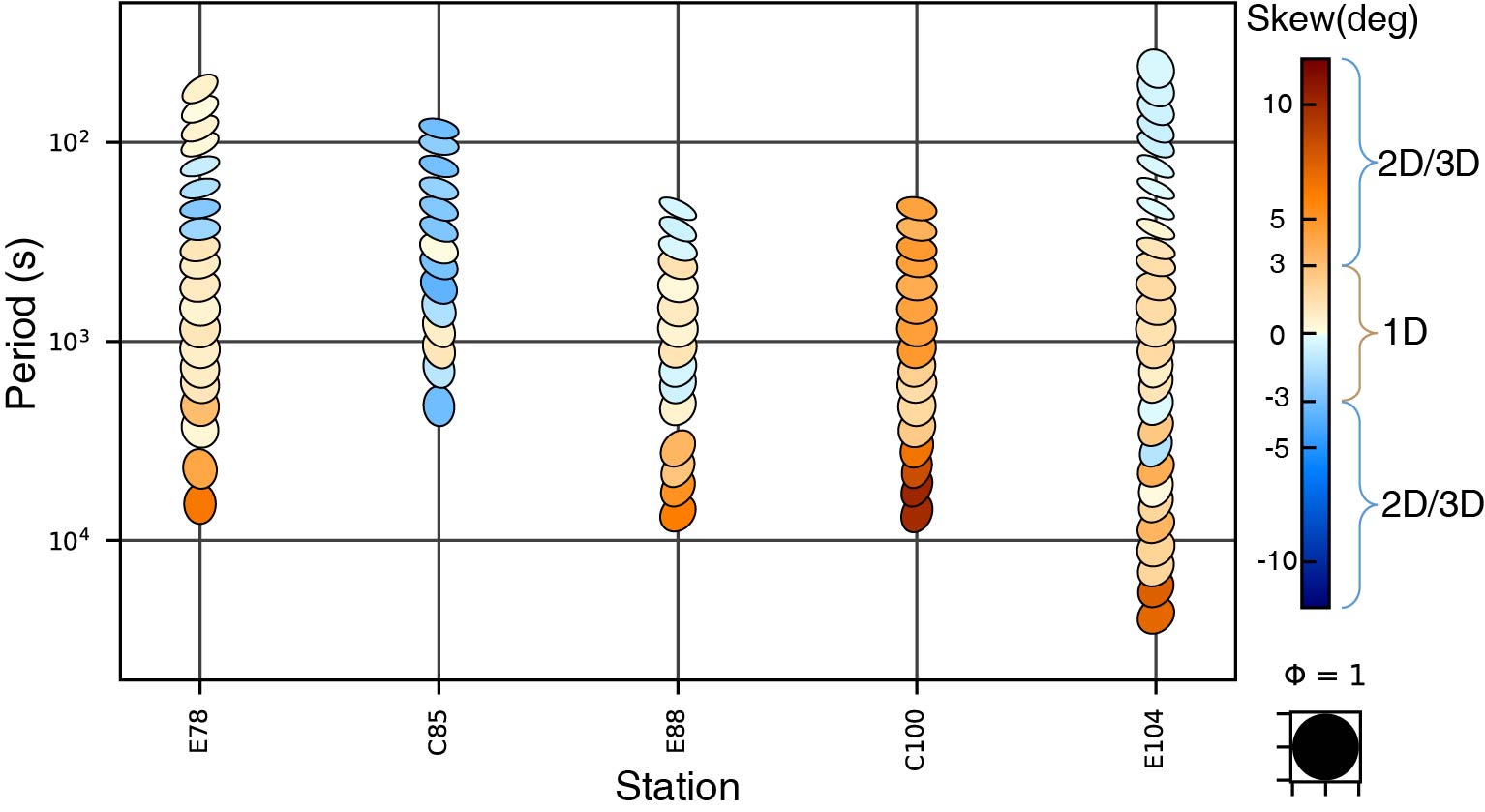


**Figure S5.** Dimensionality analyses for sites E78, C85, E88, C100, and E104. Sites C85 and C100 primarily exhibit 2-D geological structure characteristics, transitioning to 3-D features when the period exceeds 3400 seconds. For periods of 500-2100 s, E78, E88, and E104 display 1-D characteristics, while periods outside this range exhibit 2-D structures, transitioning toward 3-D structures at more extended periods.

**Rotation invariant TE and TM**

For a 2-D structure, if the data collection coordinates of the MT method are not aligned with the geological strike, a rotation of the axes is needed to recover the standard transverse electric (TE) and transverse magnetic (TM) modes. In an ideal 2-D case, the diagonal elements should be zeros. We utilized two rotation invariants to compact the four elements of the tensor into just the TE and TM impedances. The method ensures that the diagonal elements become zero, as in an ideal 2-D case, by solving a quadratic equation [9]. The invariant impedances were recovered as complex resistivities $\rho_{\pm}$ through the equation:

$\rho_{\pm}=\rho_{s}\pm\sqrt{\rho_{s}^{2}-\rho_{s}\rho_{p}\varepsilon^{2}}$. (S1)

Where the series resistivity $\rho_{s}=\frac{1}{2}(\frac{1}{\omega\mu_{0}})Z_{s}^{2}$, and the parallel resistivity $\rho_{p}=2(\frac{1}{\omega\mu_{0}})Z_{p}^{2}$. The factor ε^2^ is a function of the shear parameter [10] and can be assumed to be 1 for marine MT data [11]. The series and parallel impedances can be obtained through the four elements of the tensor $Z_{xx}$, $Z_{yy}$,$Z_{xy},Z_{yx}$ [12]:

$Z_{s}^{2}=Z_{xx}^{2}+Z_{yy}^{2}+Z_{xy}^{2}+Z_{yx}^{2}$, (S2)

$Z_{p}^{2}=\frac{{(Z_{xx}Z_{yy}-Z_{xy}Z_{yx})}^{2}}{Z_{xx}^{2}+Z_{yy}^{2}+Z_{xy}^{2}+Z_{yx}^{2}}$. (S3)

The corresponding phases of $\rho_{\pm}$ are as follows:

$\phi_{\pm}=\frac{1}{2}{tan}^{-1}\frac{Im(\rho_{\pm})}{Re(\rho_{\pm})}.$ (S4)

This method allows us to calculate the invariant TE and TM for sites C85, E88, and C100 consistently. It has been proven effective even in inconsistent strike directions between frequencies or when there is no information on the instrument orientation due to compass failure [11,13]. Fig. S6 shows the comparison before and after impedance rotation. The rotation invariant method indicates that by setting the diagonal elements to zero, the amplitude of the off-diagonal elements is enhanced, especially at low frequencies.


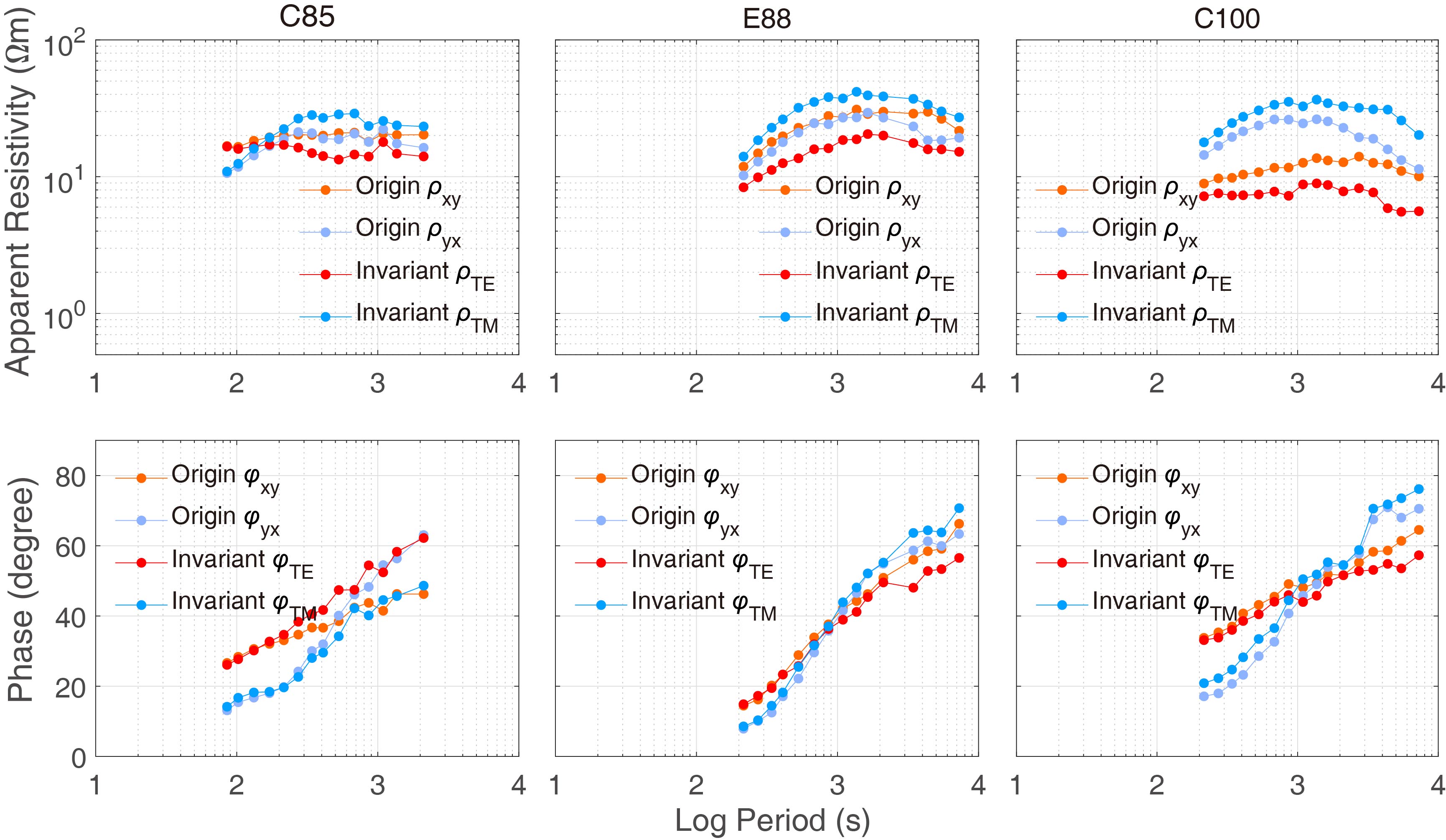


**Figure S6.** Comparisons of apparent resistivity and phase between the xy and yx directions in the magnetic north coordinate system and the rotation invariant TE and TM modes.

**MT data inversion and uncertainty quantification**

The Occam inversion method [14] was applied to perform 1-D inversion on the five sites. This 1-D inversion has been widely used in studying 2-D features like mid-ocean ridges [15,16]. Uncertainty quantification was conducted using the recently developed trans-dimensional Markov chain Monte Carlo (MCMC) algorithm [17,18].

Initial geological models were established based on the seismically determined crustal structures in the study area. The average water depth in the study area is nearly 4000 m, and the seawater was divided into 20 layers for each model, with the resistivity fixed at 0.3 Ω m. Below the seawater layer is a layered half-space with an initial resistivity of 1 Ω m, set to be free parameters during the inversion. The rotation invariant TE was used for sites C85, E88, and C100, while E78 and E104 utilized TE mode data that had undergone impedance rotation to align with the structural direction. The error floors for apparent resistivity and phase data were set to 5% and 1.43°, respectively.

Fig. S7 illustrates the variation in apparent resistivity of the TE polarization mode with depth during the inversion iteration process for each site. Fig. S8 shows that observed curves fit well with the inversion model curves.


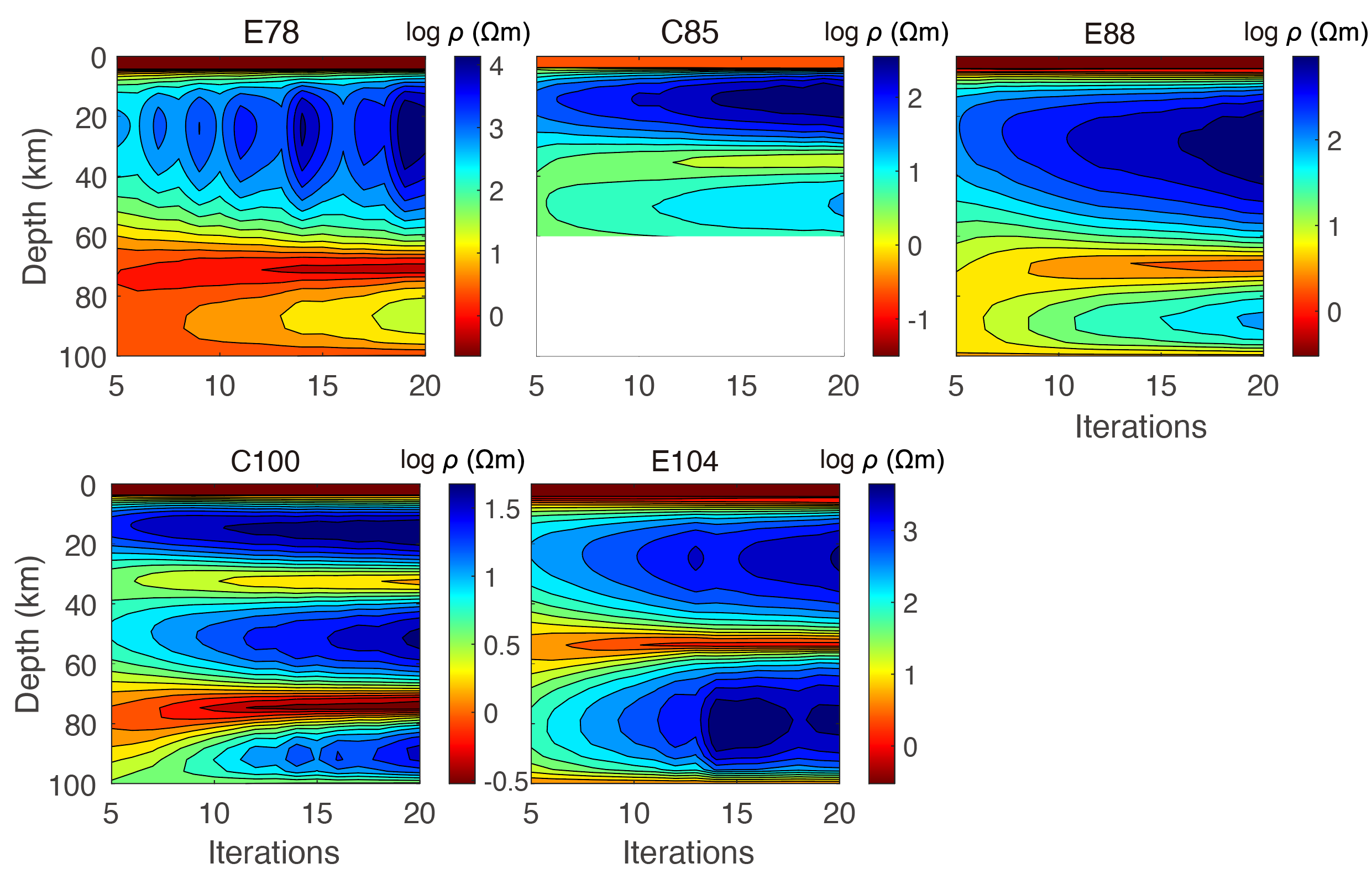


**Figure S7.** Apparent resistivity of the TE mode varies with inversion iterations.


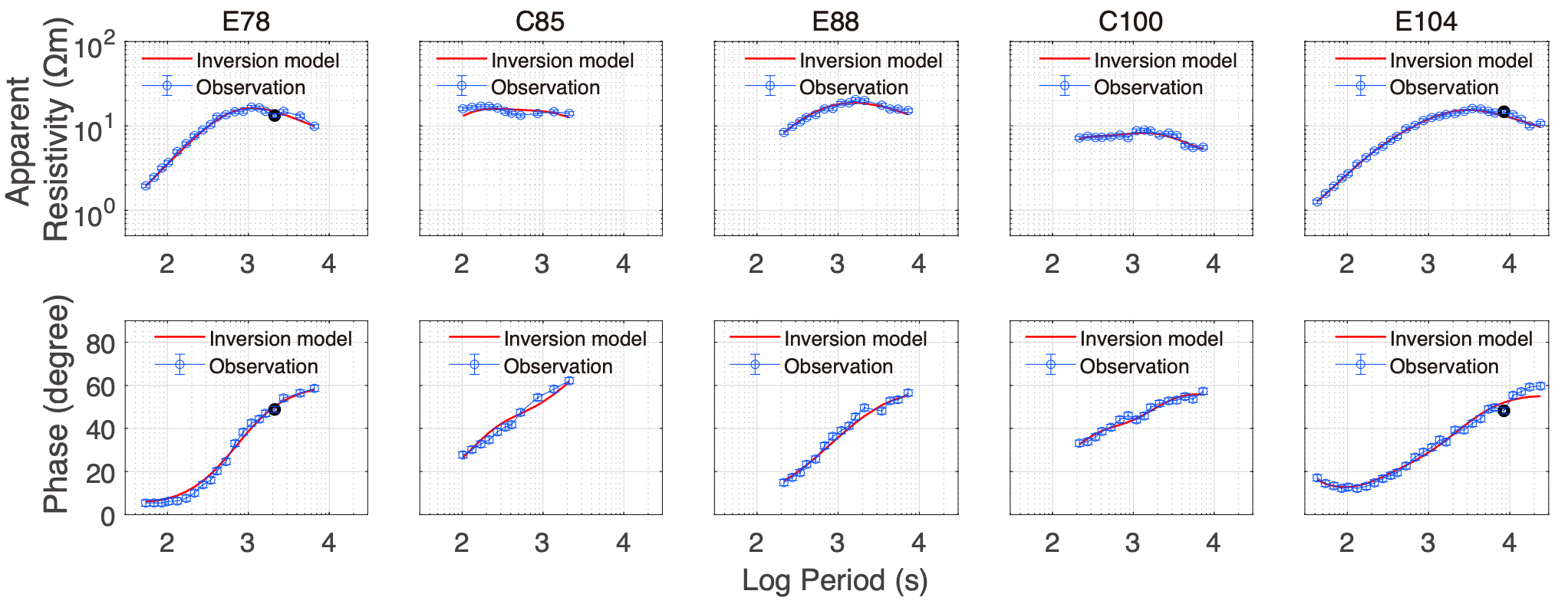


**Figure S8.** Fitting curves of the TE mode between the observation data and the inversion model. The Occam algorithm seeks to minimize a regularized functional by constraining model roughness during data fitting [14]. This regularization effectively mitigates overfitting, prevents unrealistic abrupt changes in the inverted resistivity model, and reduces the influence of data noise. As a result, the inversion yields a smooth electrical resistivity structure rather than a strict point-by-point match to the data. Overall, the predicted resistivities and phases align well with observations, with the root mean squares of 1.13–1.8 (Fig. S9). Black circles at sites E78 and E104 highlight points with large discrepancies in the inverted and observed resistivity and phase values. Further discussion on the points could be found in Fig. S9.

We adapted a trans-dimensional MCMC algorithm to invert MT data and conducted uncertainty quantification analyses on the inversion results [17]. The number of iterations was set to 1e6, with 8 chains, to ensure the accuracy and stability of the results. We overlaid the regularization inversion result from the Occam method with the probability inversion result from the trans-dimensional MCMC method, as shown in Fig. 2.

Although our primary focus is on along-axis variations, we also inverted TM and rotational invariants based on the square root of the determinant and the sum of squared elements of the tensor MT response for comparison (Fig. S9), following Baba et al. [19] and Rung-Arunwan et al. [20] .


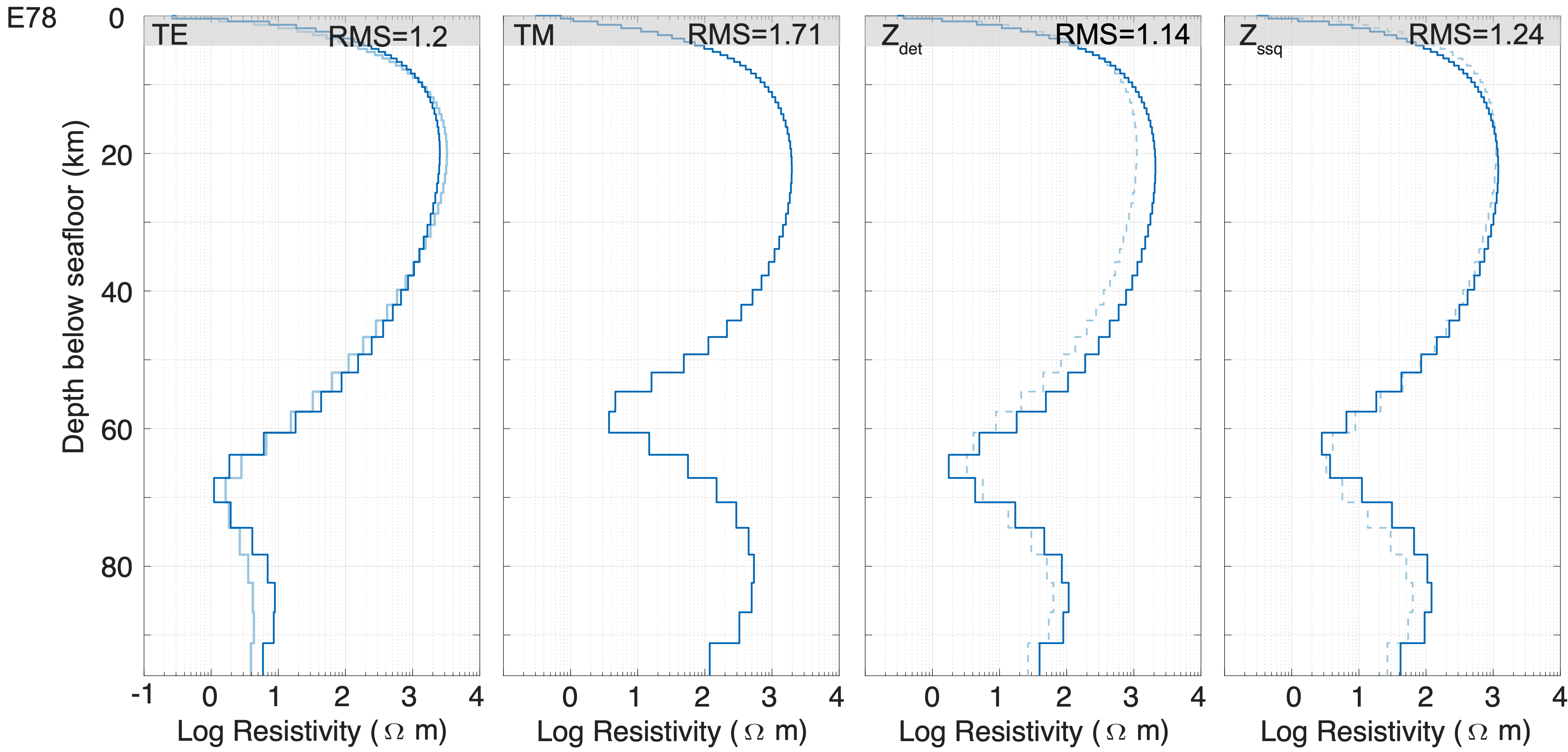


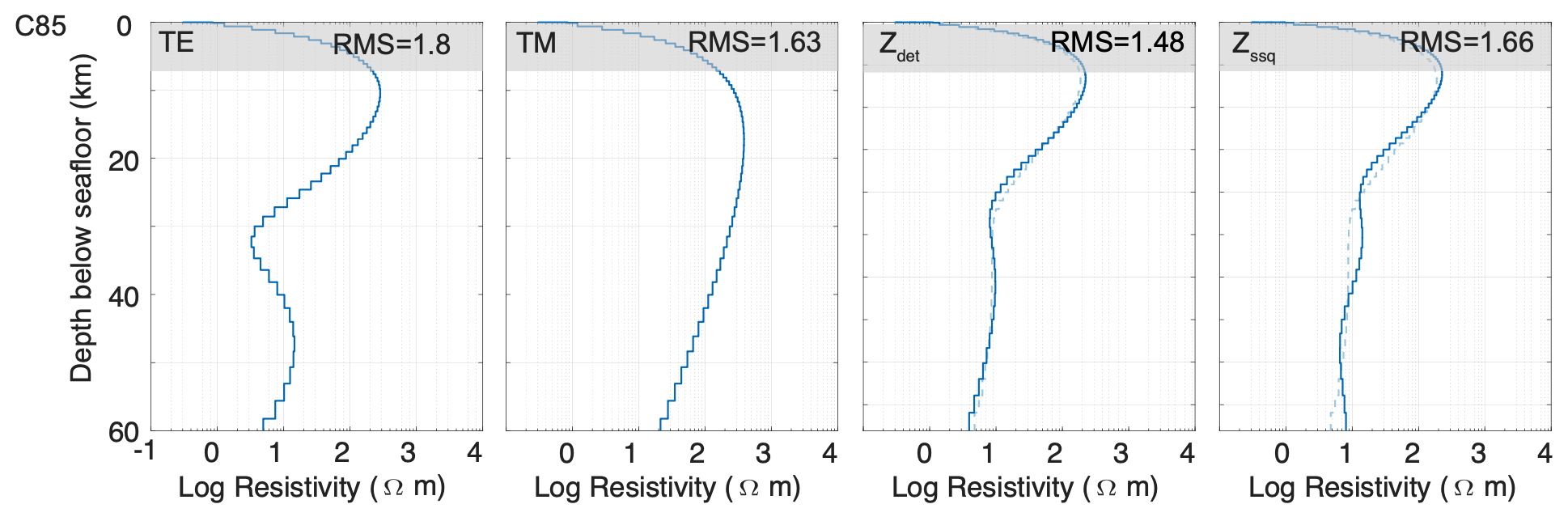


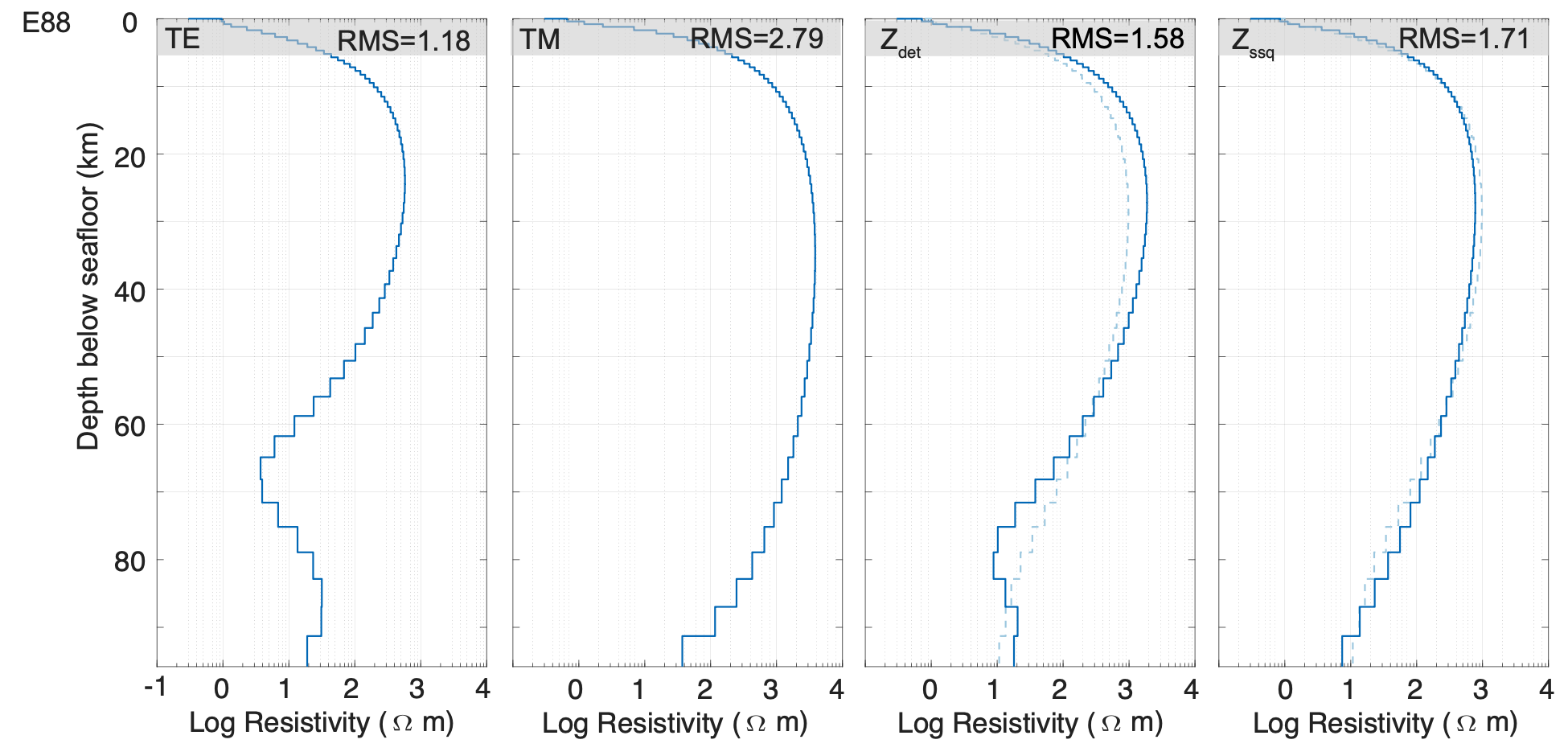


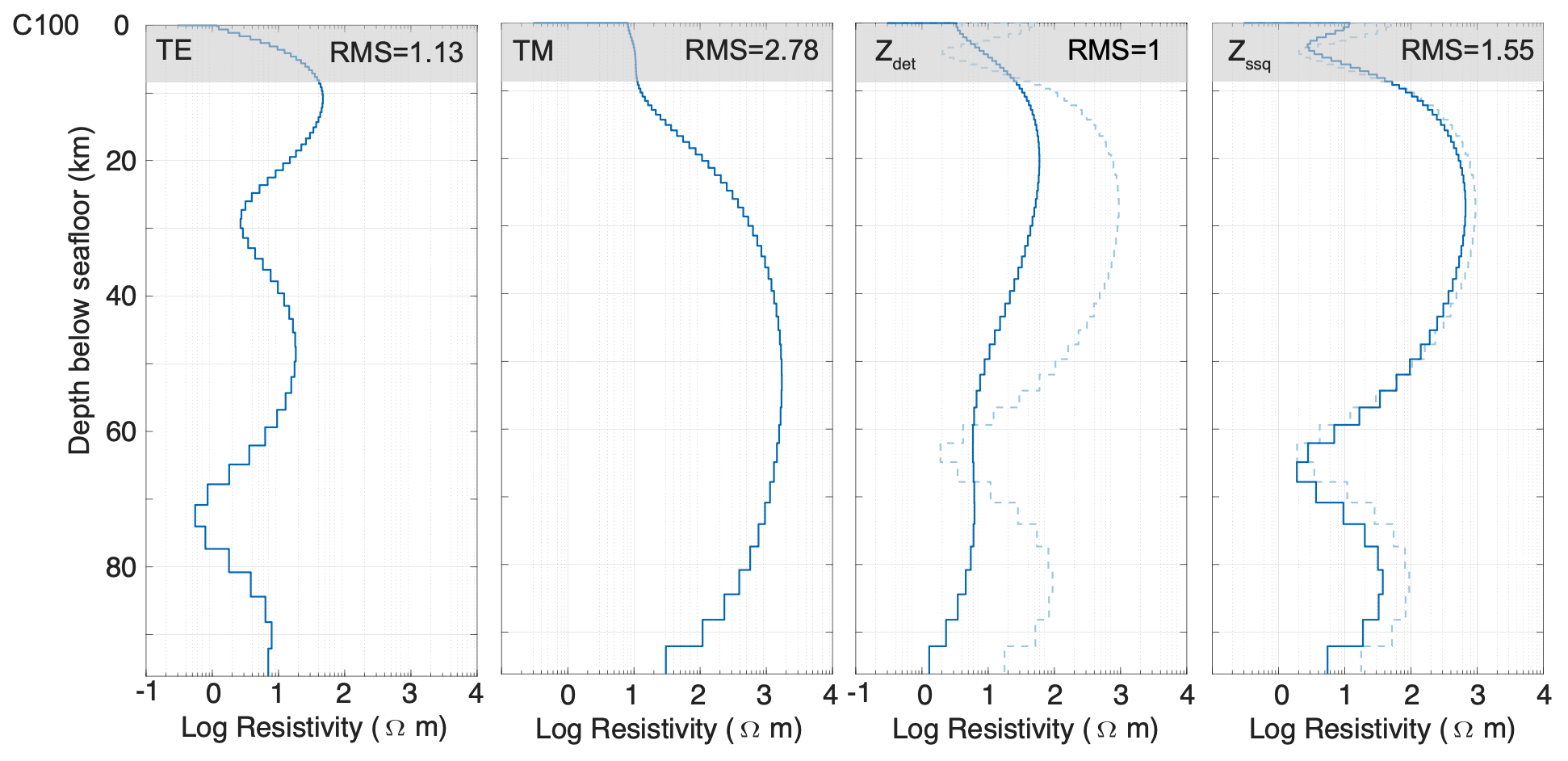


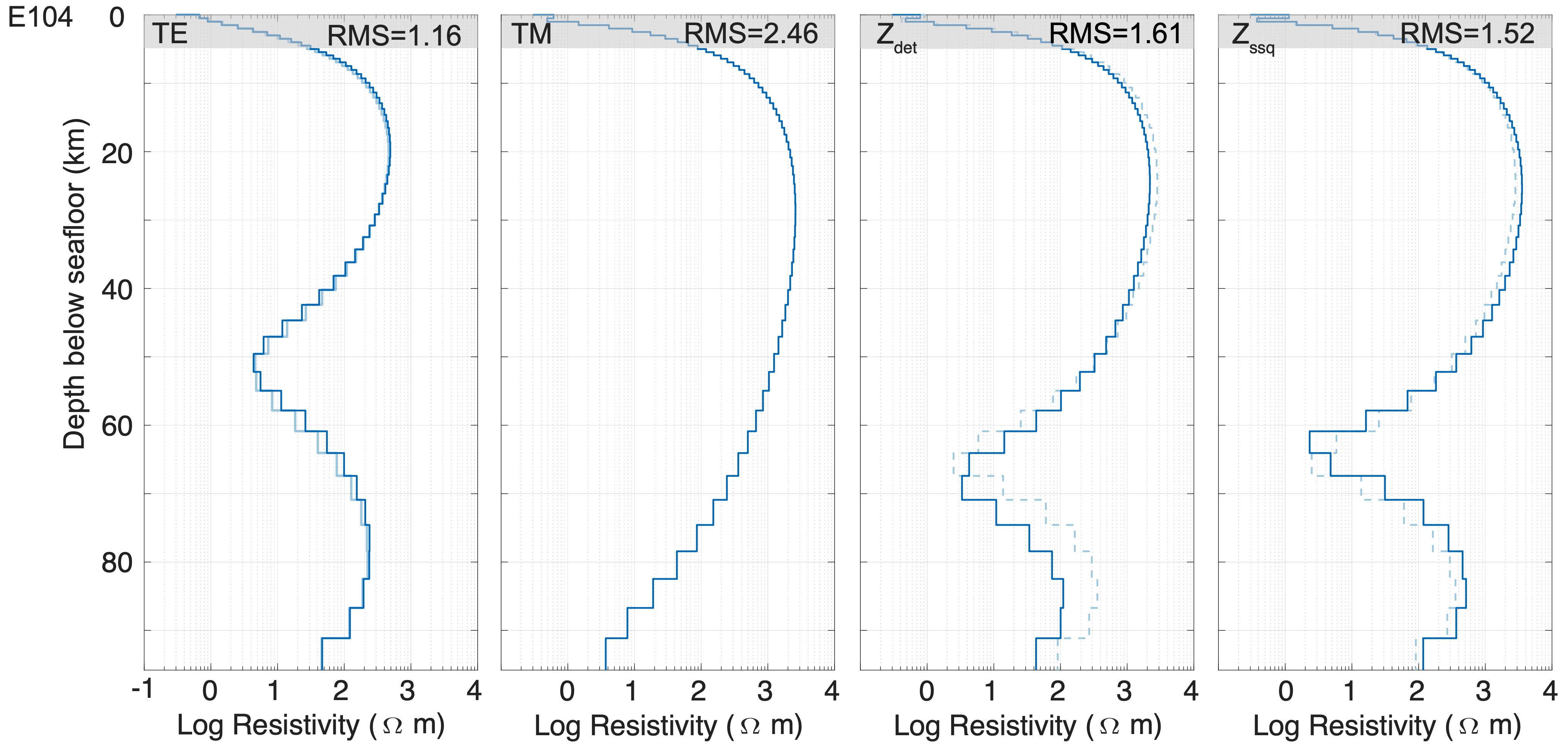


**Figure S9.** TE, TM, and rotational invariants based on the square root of the determinant (Zd_et_) and the sum of squared elements (Z_ssq_) of the tensor MT response. The averages of TE and TM are also shown with dashed lines for comparation. Note that TE (parallel to the ridge axis) generally exhibits lower resistivity, whereas TM (perpendicular to the ridge axis) tends to have higher resistivity. This trend is consistent with the apparent resistivity and phase data (Fig. S10) and aligns with the understanding that the lithosphere of ultraslow-spreading ridges cools rapidly after moving off-axis. We removed the data points (marked by black circles in Fig. S8) at E78 and E104 that showed large discrepancies in resistivity or phase between the predicted and observed values, then restarted the inversion. The updated results (light blue lines) closely align with the original ones, confirming the robustness of the initial inversion.

**From electric structure to mantle melting**

In the inversion results, a depth of 100 Ω m is designated as the eLAB, following the definition of Johansen *et al.* [21]. The crustal structure and the depth of the Moho are determined by seismic and gravity data [22]. The theoretical thermal structure along the ridge axis is derived from a combination of conductive cooling and adiabatic upwelling models [21,23]. This theoretical temperature profile is further constrained by MT inversion data and the resistivity-temperature model for dry peridotite (SEO3) [24], as shown in Fig. S10.

Using the obtained temperature profiles, we applied a resistivity mixing model for polyphase materials (dry peridotite and molten peridotite) [25] alongside the MORB model at varying water contents [26] to estimate the degree of mantle melting beneath the Gakkel Ridge.


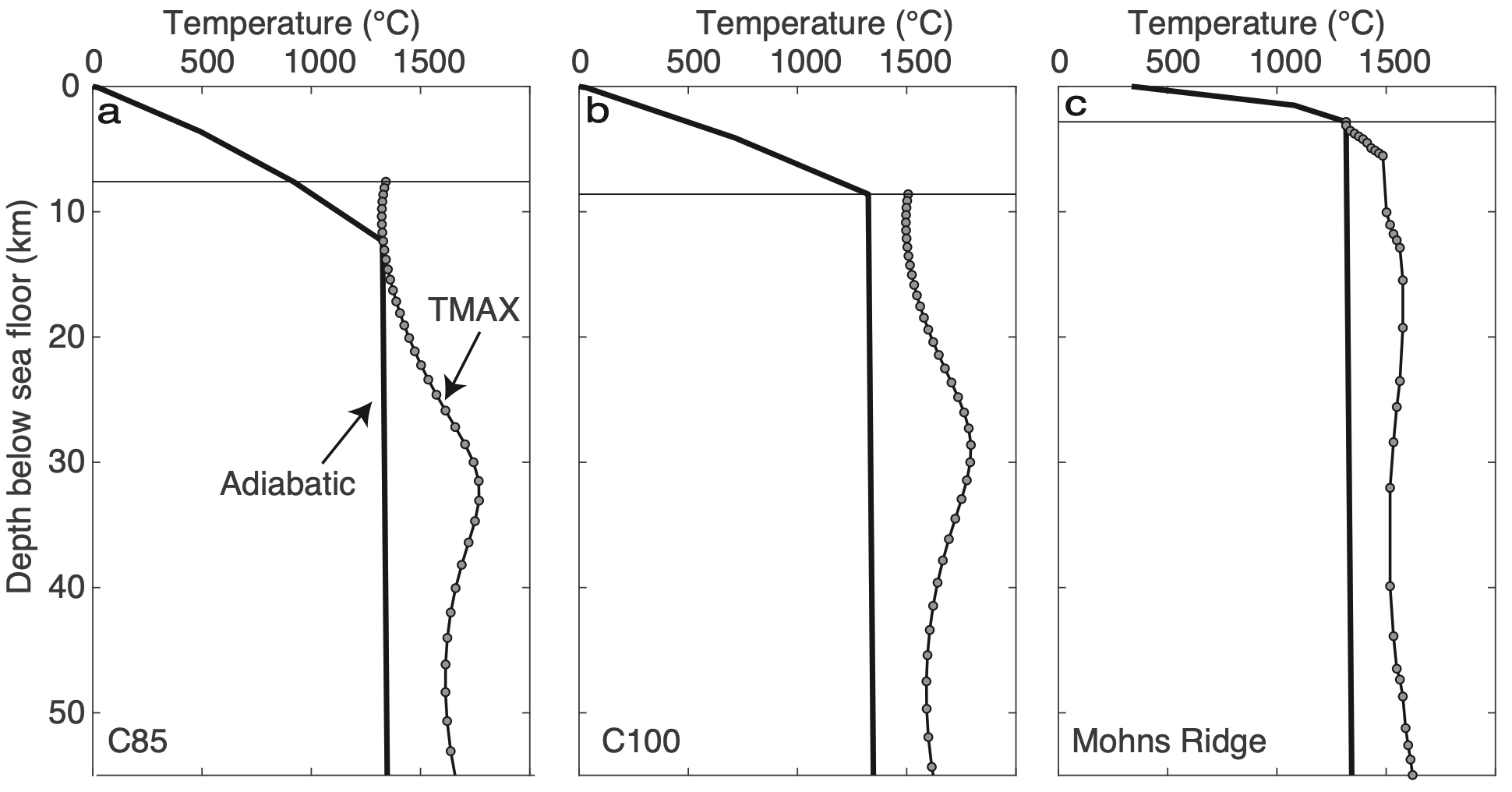


**Figure S10.** Temperature versus depth beneath the Gakkel and Mohns ridges. a, Temperature versus depth for C85. TMAX, derived by inverting measured conductivities to T_SEO3_ [24], provides the upper limit of mantle temperatures (grey circles). The temperature profile utilized in this study (heavy black line) is consistent with the adiabatic upwelling combined with a half-space cooling model. b, Temperature versus depth for C100. c, Temperature versus depth for the Mohns Ridge [21].


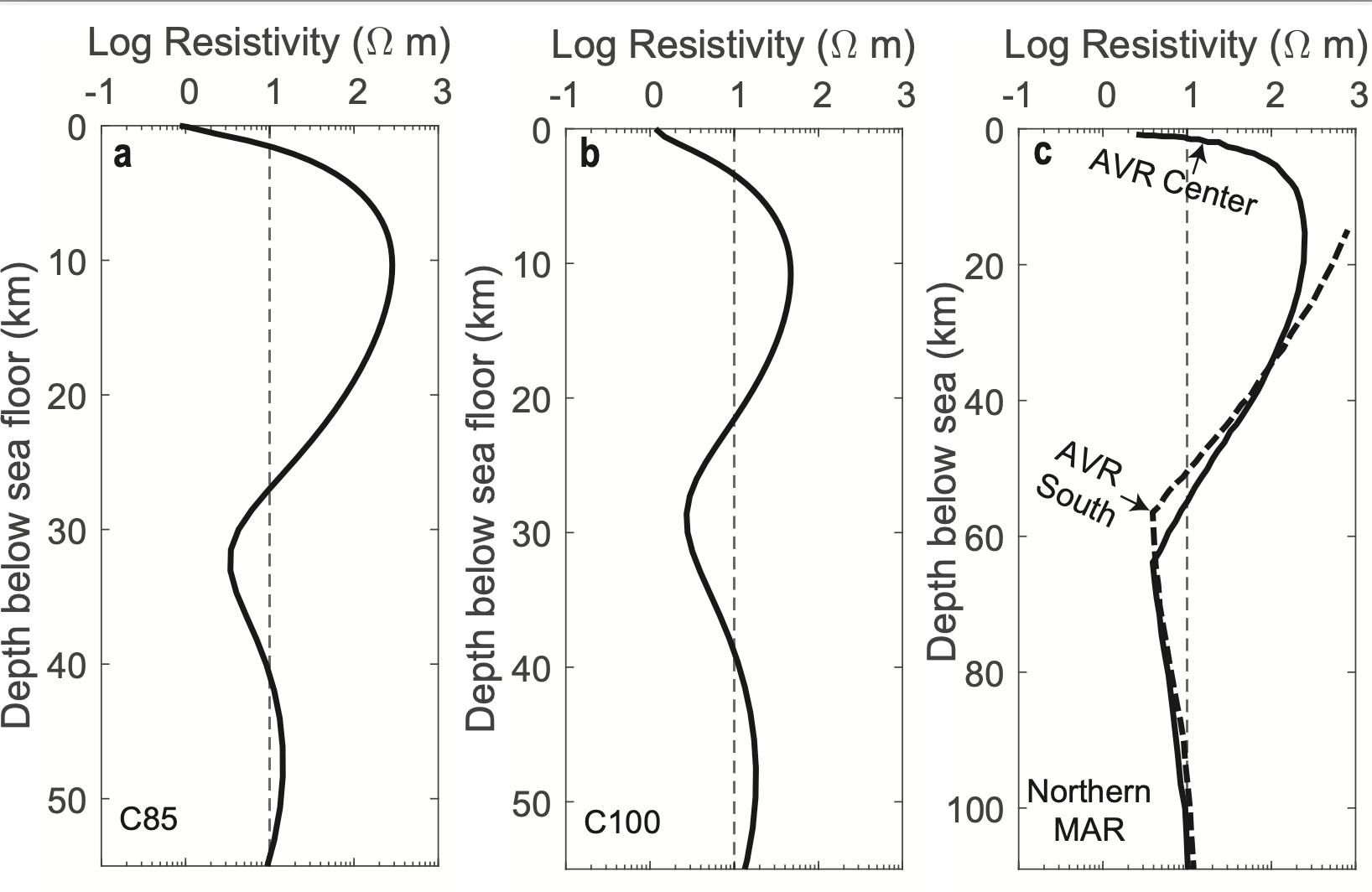


**Figure S11.** Comparisons of electrical resistivities at sites C85 (a), C100 (b), and the northern Mid-Atlantic Ridge (c) [15]. AVR, axial volcanic ridge.

## **References**

1. Chen K, Wei W-B, Deng M *et al.* A seafloor electromagnetic receiver for marine magnetotellurics and marine controlled-source electromagnetic sounding. *Appl Geophys* 2015;**12**:317–26.

2. Egbert GD. Robust multiple-station magnetotelluric data processing. *Geophys J Int* 1997;**130**:475–96.

3. Hill GJ, Wannamaker PE, Maris V *et al.* Trans-crustal structural control of CO2-rich extensional magmatic systems revealed at Mount Erebus Antarctica. *Nat Commun* 2022;**13**:2989.

4. Caldwell TG, Bibby HM, Brown C. The magnetotelluric phase tensor. *Geophys J Int* 2004;**158**:457–69.

5. Kirkby A, Zhang F, Peacock J *et al.* The MTPy software package for magnetotelluric data analysis and visualisation. *J Open Source Softw* 2019;**4**:1358.

6. Bibby HM, Caldwell TG, Brown C. Determinable and non‐determinable parameters of galvanic distortion in magnetotellurics. *Geophys J Int* 2005;**163**:915–30.

7. Simpson F, Bahr K. Practical Magnetotellurics. 2005, DOI: 10.1017/cbo9780511614095.

8. Booker JR. The Magnetotelluric Phase Tensor: A Critical Review. *Surv Geophys* 2014;**35**:7–40.

9. Gómez-Treviño E, Romo JM, Esparza FJ. Quadratic solution for the 2-D magnetotelluric impedance tensor distorted by 3-D electro-galvanic effects. *Geophys J Int* 2014;**198**:1795–804.

10. Groom RW, Bailey RC. Decomposition of magnetotelluric impedance tensors in the presence of local three‐dimensional galvanic distortion. *J Geophys Res* 1989;**94**:1913–25.

11. Montiel-Álvarez AM, Romo JM, Constable S *et al.* Invariant TE and TM impedances in the marine magnetotelluric method. *Geophys J Int* 2020;**221**:163–77.

12. Gómez-Treviño E, Antonio-Carpio R, Romo JM *et al.* A recursive set of invariants of the magnetotelluric impedance tensor. *Acta Geod Geophys* 2013;**48**:265–74.

13. Gómez-Treviño E, Muñiz Y, Cuellar M *et al.* Invariant TE and TM magnetotelluric impedances: application to the BC87 dataset. *Earth, Planets Space* 2018;**70**:133.

14. Constable SC, Parker RL, Constable CG. Occam’s inversion; a practical algorithm for generating smooth models from electromagnetic sounding data. *Geophysics* 1987;**52**:289–300.

15. Heinson G, Constable S, White A. Episodic melt transport at mid‐ocean ridges inferred from magnetotelluric sounding. *Geophys Res Lett* 2000;**27**:2317–20.

16. Heinson G, Constable S, White A. Seafloor magnetotelluric sounding above axial seamount. *Geophys Res Lett* 1996;**23**:2275–8.

17. Blatter D, Key K, Ray A *et al.* Bayesian joint inversion of controlled source electromagnetic and magnetotelluric data to image freshwater aquifer offshore New Jersey. *Geophys J Int* 2019;**218**:1822–37.

18. Blatter D, Morzfeld M, Key K *et al.* Uncertainty quantification for regularized inversion of electromagnetic geophysical data—Part I: motivation and theory. *Geophys J Int* 2022;**231**:1057–74.

19. Baba K, Utada H, Goto T *et al.* Electrical conductivity imaging of the Philippine Sea upper mantle using seafloor magnetotelluric data. *Phys Earth Planet Inter* 2010;**183**:44–62.

20. Rung-Arunwan T, Siripunvaraporn W, Utada H. On the Berdichevsky average. *Phys Earth Planet Inter* 2016;**253**:1–4.

21. Johansen SE, Panzner M, Mittet R *et al.* Deep electrical imaging of the ultraslow-spreading Mohns Ridge. *Nature* 2019;**567**:379–83.

22. Zhang T, Li J, Niu X *et al.* Highly variable magmatic accretion at the ultraslow-spreading Gakkel Ridge. *Nature* 2024;**633**:109–13.

23. Turcotte D, Schubert G. Geodynamics. 2014, DOI: 10.1017/cbo9780511843877.

24. Constable S. SEO3: A new model of olivine electrical conductivity. *Geophys J Int* 2006;**166**:435–7.

25. Hashin Z, Shtrikman S. A Variational Approach to the Theory of the Effective Magnetic Permeability of Multiphase Materials. *J Appl Phys* 1962;**33**:3125–31.

26. Ni H, Keppler H, Behrens H. Electrical conductivity of hydrous basaltic melts: implications for partial melting in the upper mantle. *Contrib Miner Pet* 2011;**162**:637–50.
